# Longitudinal Weight Gain and Related Risk Behaviors during the COVID-19 Pandemic in Adults in the US

**DOI:** 10.3390/nu13020671

**Published:** 2021-02-19

**Authors:** Surabhi Bhutani, Michelle R. vanDellen, Jamie A. Cooper

**Affiliations:** 1 School of Exercise and Nutritional Sciences, San Diego State University, ENS Building, Room 302 5500 Campanile Drive San Diego, San Diego, CA 92182, USA; 2 Department of Psychology, University of Georgia, Athens, GA 30602, USA; mvd@uga.edu; 3 Department of Foods and Nutrition, University of Georgia, Athens, GA 30602, USA; jamie.cooper@uga.edu

**Keywords:** body weight, eating behaviors, physical activity, sedentary activity, psychological factors

## Abstract

Cross-sectional analyses have shown increased obesogenic behaviors and a potential for weight gain during COVID-19 related peak-lockdown (March–May 2020), but longitudinal data are lacking. This study assessed longitudinal changes in body weight and lifestyle behaviors in the US adults during the pandemic. Methods: We used Qualtrics survey to collect self-reported data on body weight, dietary, physical activity, and psychological variables (*n* = 727) during the peak-lockdown (April/May) and at post-lockdown (September/October). Peak-lockdown weight data were categorized based on the magnitude of weight gained, maintained, or lost, and behavioral differences were examined between categories at two time points. Results: Body weight increased (+0.62 kg; *p* < 0.05) at the post-lockdown period. The body mass index also increased (26.38 ± 5.98 kg/m^2^ vs. 26.12 ± 5.81 kg/m^2^; *p* < 0.01) at the post-lockdown period vs. peak-lockdown period. Close to 40% of participants reported gaining either 1–4 lbs or >5 lbs of body weight during the peak-lockdown, while 18.2% lost weight. Weight-gainers engaged in riskier dietary behaviors such as frequent ultra-processed food intake (*p* < 0.01) and snacking (*p* < 0.001), were less active, and reported high stress and less craving control during peak-lockdown. Of those gaining >5 lbs, 33% continued to gain weight after the lockdown eased, while 28% maintain higher body weight. In weight-gainers, takeout meal frequency increased, and high ultra-processed food intake and stress, and low craving control continued to persist after the lockdown eased. Conclusion: We show that the COVID-19 lockdown periods disrupted weight management among many Americans and that associated health effects are likely to persist.

## 1. Introduction

The SARS-CoV-2 virus, responsible for the Coronavirus (COVID-19) pandemic, has become a threat to public health in the United States. While the initial shelter-in-place mandate (March through May 2020) helped curb the spread of this virus, the mandate also carries long-term implications for other health behaviors. One concern that evolved during the pandemic was the fear of gaining excessive body weight, frequently referred to on social media as “Quarantine 15”, “gaining the COVID-19”, or “fattening the curve”. Supporting these speculations, early investigations of a cross-sectional dataset showed that 22% of adults self-reported gaining 5–10 lbs of body weight during self-quarantine [1]. The current situation of extended lockdown is similar to but longer than the winter holiday period (6–8 weeks), which contributes to a modest average weight gain of 0.5 kg [2]. This seemingly small change in body weight during the holidays contributes to more than half of annual weight gain in adults. More importantly, this weight is not subsequently lost [2,3,4,5,6] and can lead to a substantial increase over multiple decades. If weight gain during the pandemic follows the same trajectory as weight increases reported during the holiday period, pandemic-related weight gain will be an additional major contributor to the annual weight gain in the year 2020 and possibly even in 2021. Moreover, the lasting effects of the COVID-19 pandemic on weight gain are unclear, thus require thorough scrutiny.

Pandemic-related disruptions to daily routines have impacted multiple behavioral measures, particularly during the peak of the lockdown period [1,7,8,9]. Peak lockdown is described as the time period between March and May 2020, when most countries required people to stay at home, and ordered most workplaces and public services to close down. In a recent analysis of cross-sectional data collected during the peak lockdown months, we and others reported alterations in health behavior measures [1,7,8,9,10], raising concerns for a possible long-term impact of this pandemic on energy balance. In particular, physical activity (PA) and self-weighing frequency declined, while snacking, energy-dense and healthy food consumption, and sedentary activity all increased in our participants [8,10]. Sleeping patterns, sleep quality, and mood also shifted during the pandemic in a direction that supports weight gain [8]. Similar to the holiday period, these short-term alterations in energy intake and PA behavioral norms may accelerate weight gain, possibly contributing to long-term changes in body weight. Considering that 78% of American adults are projected to be overweight or obese by year 2030 [11], and that fluctuation in health behaviors drives poor energy intake and energy expenditure, examining shifts in health behaviors during the recently extended lockdown is important for understanding the broader public health impacts of the COVID-19 pandemic.

To assess the longitudinal impacts of the COVID-19 pandemic on health behaviors, we quantified the change in self-reported body weight by adults in the U.S. during the initial shelter-in-place recommendations implemented in April/May 2020 and at a 5-month follow-up in September/October 2020. We also quantified habitual dietary behaviors, PA and sedentary behaviors, and psychological variables during these two periods. Specifically, we sought to understand how health behaviors and psychological factors fluctuate long-term in individuals reporting varying degrees of weight gain/weight loss during the peak-lockdown period. These analyses are helpful in identifying acute changes in obesogenic health behaviors, as well as potential recovery from (or lack thereof) disruptions to health behaviors incurred during the pandemic.

## 2. Materials and Methods

### 2.1. Study Design and Participants

We collected self-reported longitudinal data where a convenience sample of U.S. adults completed an online survey delivered using Qualtrics (Qualtrics^®^ Software Company Provo, UT, USA and Seattle, WA, USA). All participants provided online consent before completing the questionnaire. The Qualtrics questionnaire was administered through either Amazon Mechanical Turk (Mturk) or via social media, email, and word of mouth. Mturk (© 2005–2018, Amazon Mechanical Turk, Inc., Seattle, WA, USA) is a web service that enables researchers to survey the target population across the US. Prior studies show that U.S. workers on Mturk are similar to the U.S. population and provide diversity in terms of age, ethnicity, and socioeconomic status, and can provide valid and reliable results for health research [12,13]. A total of 1779 men (43.38%) and women (56.62%) between the age of 18 and 75 years completed the survey during the peak lockdown period (24 April to 4 May), while shelter-in-place guidelines were instituted widely across the country. We refer to this time point as the “peak-lockdown period”. A follow-up survey was sent to the same participants after 5 months (21 September to 13 October) via the Mturk platform or email. A total of 764 participants returned the completed survey (Mturk *n* = 586; social media/email/word of mouth *n* = 168). We refer to this second time point as “post-lockdown period”. Inclusion criteria for participation in the study included (1) access to the internet, and (2) living in the US. The Institutional Review Board at San Diego State University approved the study.

### 2.2. Questionnaire

The questionnaire included the following 6 item categories: demographics, weight behaviors, sleep and other health behaviors, eating behaviors, PA behaviors, and psychological factors. Questions within these categories aimed to collect data on practices and beliefs during the peak-lockdown period and post-lockdown follow-up at 5-months. The survey took approximately 25 min to complete.

### 2.3. Measures

**Figure 1:**
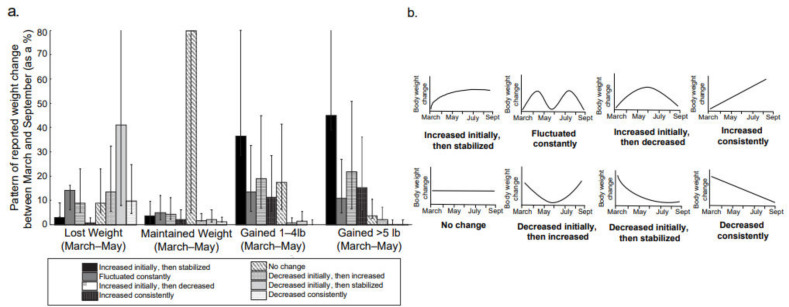
Pattern of Reported Weight Change Between March and September. (**a**) Percent of participants reporting patterns of body weight fluctuation across weight risk groups. (**b**) Options of patterns presented to participants to describe their weight change between March and September. Error bars represent 95% Confidence Interval.

Weight Change. Self-reported body weight and height were used to determine the change in body mass index (BMI). Participants also self-reported weight change between March through May (during the peak-lockdown period), and May through October using the following options lost > 10 lbs, lost 5–10 lbs, lost 1–4 lbs, maintained weight, gained 1–4 lbs, gained 5–10 lbs, gained > 10 lbs. To assess weight change perception, we asked participants how the fit of their clothes had changed since the lockdown started in March using a 10-point visual analog scale (VAS) (0 = Extremely loose, 5 = No change at all, 10 = Extremely tight/snug). Finally, the perception of weight fluctuation pattern was assessed using visual representations of weight fluctuation developed for this study as presented in Figure 1b.

Eating Behavior. Participants reported current consumption frequency of fruits, vegetables, non-diet beverages, including all sugar-sweetened beverages (SSB; for example, Coke, Pepsi, flavored juice drinks, sports drinks, sweetened teas, coffee drinks, energy drinks, electrolyte replacement drinks), diet beverages, restaurant/takeout/fast food/delivery food, and processes and ultra-processed foods (using the NOVA classification system categories) [14]. Response options were: Never, Less than once a month, Less than once a week, Once a week, Several times a week, Once a day, Twice a day, Three or more times a day.

Physical Activity (PA). PA measures were determined as described previously [8]. Briefly, we used the validated [15,16] International Physical Activity Questionnaire (IPAQ short version) [17] to determine metabolic equivalents (METs) during the two time points. Amount of time spent watching television (TV; including videos on DVD, Blue Ray, Netflix, Hulu, Amazon Prime, etc.) and time spent on other leisure screen time activities (such as a computer, video games, email, Facebook, Instagram, Twitter, etc.) was also collected at both time points using self-reports (scale points = None, Less than 1 h, 1–2 h, 2–3 h, 3–4 h, 4 or more hours).

The Control of Eating Questionnaire (CoEQ). The validated CoEQ comprised 21 items and included questions on general appetite and overall mood (independent of craving), frequency and intensity of general food craving, craving for specific foods (e.g., dairy, starchy, sweet, or non-sweet foods), and individuals’ perceived control over resisting craved food items. Participants responded about their experience over the previous seven days. These items were assessed using a 10-point VAS. As described by Dalton et al., the following subscales were created from the questionnaire: craving control, positive mood, craving for sweet, craving for savory [18].

Sleep Duration and Stanford Sleepiness Scale (SSS). Participants were asked to report the average number of hours spent sleeping per day to assess sleep duration. Sleep quality was determined using the SSS [19], which uses a 7-point Likert scale to quantify a participant’s sleepiness at the moment with higher values indicating greater sleepiness. Participants reported their sleepiness level immediately after waking in the morning.

Multidimensional State Boredom Scale. The Multidimensional state boredom scale [20] uses eight items to assess boredom, which was reframed to measure boredom during the pandemic (e.g., Time is passing by slower than usual). Participants responded to all items on a scale from 1 (strongly disagree) to 7 (strongly agree).

### 2.4. Data Analysis

SAS version 9.4 (Cary, NC) was used for statistical analysis, and significance was set at *p* < 0.05. We first compared health behaviors during peak-lockdown and post-lockdown using repeated measures t-tests. We next considered how health behaviors might have varied across time among people who maintained versus gained (or lost) weight. For these analyses, we identified four groups based on weight change during the lockdown (lost weight, maintained weight, gained a small amount of weight (1–4 lbs), gained a large amount of weight (>5 lbs). Then, we conducted a series of analyses to evaluate whether these groups differed in health behaviors and psychological states across time (peak-lockdown, post-lockdown). For each outcome, we first conducted a 2 (time: peak-lockdown period, post-lockdown period) × 4 (risk group: lost weight, maintained weight, gained 1–4 lbs, gained > 5 lbs) mixed ANOVA (time was treated as a within-subjects and group were modeled as a between-subjects factor). We followed these tests with two sets of follow-up analyses. First, we evaluated the difference between risk groups at each time was using a one-way ANOVA (and contrast tests using Tukey’s post-hoc correction to compare all groups within time point). Second, we evaluated whether each group differed in the health behavior (or psychological state) over time using a repeated measures t-test within group. Although we conducted multiple statistical tests, they do not add to type 1 error due to multiple comparisons. These tests instead lead to convergence and are evidence of the robustness of our findings. Following the recommendations [21,22], we discuss our results in light of the cumulative evidence (e.g., consistency across tests).

## 3. Results

Of the total 764 respondents who completed the survey during both peak-lockdown period in April/May and at the follow-up post-lockdown period, 727 (males: *n* = 339, age 40.09 ± 12.40 yr; females: *n* = 388, age 40.66 ± 13.94 yr) were included in the final analysis. Respondents were excluded if they failed to complete the questionnaire, reported being pregnant, had a calculated BMI ≤15 or ≥57 kg/m^2^, had a missing BMI value, or reported having a medical condition that contributed to acute weight increase/loss. On average, participants were overweight (BMI: male 26.55 ± 5.34 kg/m^2^, female 26.22 ± 6.49 kg/m^2^), and consisted of whites (78.7%), Asian (10.5%), Black (6.7%), and other (4.2%) population groups. Over half of our study population (56%) worked full time, 16% worked part-time, and 17.5% were either unemployed, retired, or were full-time homemakers during the peak-lockdown period. As expected, 73.4% of the population reported working entirely from home during the peak-lockdown period, which decreased to 52% during the post-lockdown period. In contrast, 16.3% worked entirely at their place-of-work during the peak-lockdown period, which increased to 29.8%, respectively, during the post-lockdown period. These patterns suggest that daily routines were disrupted by the pandemic, and that for many people, those disruptions are persisting despite the easing of shelter-in-place mandates.

### 3.1. Change in Health Behaviors between Peak-Lockdown Period and Post-Lockdown Period

Table 1 shows the self-reported differences in all participants in eating behaviors, PA behaviors, and psychological states during the height of the lockdown (i.e., March–April 2020) vs. post-lockdown (i.e., May–September 2020). Average weight gain was reported to be 0.62 kg across all participants (peak-lockdown: 76.70 ± 19.79 kg vs. post-lockdown: 77.32 ± 20.01; *p* < 0.05) at 5-month follow-up. The body mass index (BMI) was also higher at post-lockdown compared to pre-lockdown period (26.38 ± 5.98 vs. 26.12 ± 5.81; *p* < 0.01). Although most eating behaviors did not differ between the two timepoints, overall, we observed an increase in the frequency of takeout/restaurant food but a decrease in the consumption of processed foods. We also observed a non-significant decline in alcohol intake, ultra-processed food intake, as well as vegetable intake at post-lockdown. Other healthy behaviors including frequency of self-weighing and weekly MET expenditure (particularly due to increased engagement in moderate activity) increased after the lockdown ended. Participants also reported less sitting time, less time spent in front of the television, and less leisure screen time after the lockdown eased. Finally, participants reported experiencing less boredom (*p* < 0.001) and more control over cravings (*p* < 0.001) during the post-lockdown period.

### 3.2. Change in Health Behaviors between Peak-Lockdown Period and Post-Lockdown Period by Weight Change Categories

**Figure 2:**
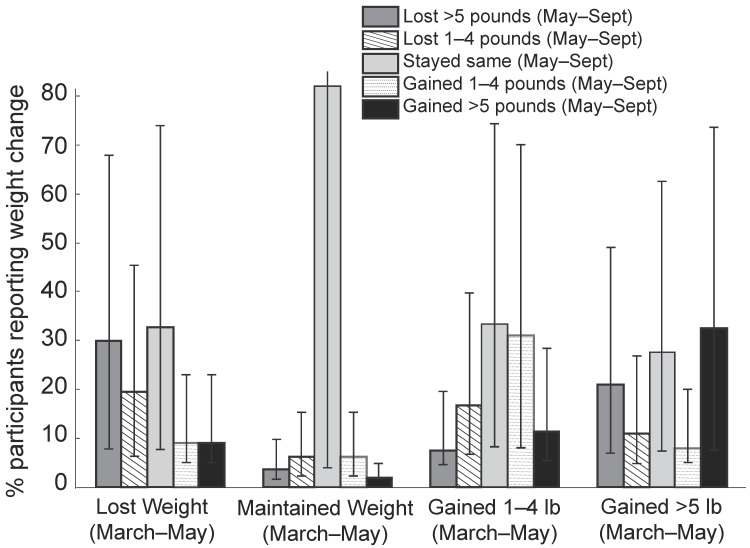
Weight change during the post-lockdown (May-September) in participants who reported to have gained, lost, or maintained body weight during the peak-lockdown period (March through May). Error bars represent 95% Confidence Interval.

Weight change. Table 2 presents weight change data in participants who reported to have gained, lost, or maintained body weight during the peak-lockdown period (March through May). Close to 40% of participants reported to have gained either 1–4 lbs or >5 lbs of body weight during this period. Of the participants (*n* = 138) who gained >5 lbs during the peak-lockdown period, over one-third (33%) continued to gain substantial weight (>5 lbs), and another other one-third (28%) continued to maintain their higher body weight, even after the lockdown was eased (Figure 2). Similarly, among participants (*n* = 132) who reported gaining 1–4 lbs during the peak-lockdown (March through May), one-third reported maintaining their weight gain after the lockdown eased, while one-third (31%) gained an additional 1–4 lbs, and 11% gained an additional 5–10 lbs after the lockdown was eased in May (Figure 2). This self-reported numerical weight gain data between May and September is supported by the data on perception of change in cloth fitting such that, as weight increased, the perception that clothes are getting tighter also increased (*p* < 0.001) in a dose-dependent manner. The BMI also reflects these findings such that BMI significantly increased in participants reporting 1–4 lbs of weight gain and >5 lbs of weight gain (Table 2). No such changes were reported in individuals who reported to either have lost or maintained body weight. Reflecting on the perceived pattern of weight change from May through September, ≈45% of participants who gained >5 lbs of body weight and ≈36% who gained 1–4 lbs reported their weight pattern to increase during initial periods of pandemic and then stabilize at that higher level (Figure 1a,b).

Dietary behaviors. Dietary behaviors varied across weight gain groups (Table 2). The most notable effects were that high weight gain groups (either 1–4 lbs or >5 lbs) reported more frequent snacking and alcohol intake during peak-lockdown and reduced it significantly during the post-lockdown follow-up. No such difference in snacking frequency were reported at post-lockdown between weight groups. Intake of ultra-processed foods was also high during peak-lockdown for both weight gain groups and continued to be high in the >5 lbs weight gain group post-lockdown. Takeout/restaurant meal frequency increased in all weight change groups during the follow-up. We also observed that weight gainers ate more fruits during the pre-lockdown period compared to post-lockdown period. The only time by group interaction to emerge was for snacking. The pattern of results for this interaction indicates that participants who lost weight during lockdown snacked less during lockdown than after it eased, whereas participants who gained weight (1–4 lbs) reported more snacking during lockdown than after it eased.

PA Behaviors. PA-related risk behaviors differed across weight groups (Table 2). Participants who gained weight (either 1–4 lbs or >5 lbs) during the peak-lockdown were more likely to report greater TV (*p* < 0.05) and leisure screen time (*p* < 0.05) than participants whose weight did not change. Weight maintainers reported less leisure screen time during peak-lockdown. The only time × group interaction suggests that TV screen time was greater during (vs. after) lockdown for weight maintainers and gainers than for participants who lost weight during lockdown. Participants who gained weight during peak-lockdown reported less energy expenditure than participants lost weight and reported engaging in less vigorous PA.

Psychological State and Other Health Variables. Table 3 presents the variation in psychological state variables across weight change groups. The higher risk groups (gaining 1–4 lbs or >5 lbs) generally reported more stress and sleepiness during the peak-lockdown period. They also reported greater boredom during this period, along with participants who lost weight. Both weight gain groups also reported less control over their cravings during the peak-lockdown, which persisted even after the lockdown eased. Most notably, the weight change groups (weight loss and weight gain) persisted with higher levels of stress after the lockdown eased. Participants who lost weight during the peak-lockdown tended to report more boredom and less positive mood than weight-maintainers. The only time by group interaction to emerge suggests that sleepiness differed across weight time during and after peak lockdown. All groups reported more sleepiness during the lockdown but the improvement from during to post-lockdown was largest among the highest weight gain group. The highest weight gain group appeared to report sleep difficulties during COVID even if their overall sleep time did not change.

## 4. Discussion

This study is the first to show longitudinal changes in body weight, BMI, and lifestyle behaviors in US adults during the COVID-19 pandemic. As predicted, our participants gained significant body weight (0.62 kg) during the pandemic, thus following a similar trend as the winter holiday weight gain, which is a major contributor to the annual body weight change. Of concern, this COVID-19-related weight increase will be an add-on to the annual holiday weight gain in the year 2020, putting people at a greater risk for metabolic disorders. Examining weight changes during the height of the lockdown period, 37.2% of our participants reported gaining body weight (19% gained >5 lbs, 18.2% gained 1–4 lbs) during that time frame (March–May). These findings are comparable to a recent report by Zachary et al. [1], where 22% of participants reported gaining >5 lbs during the peak-lockdown period. Additionally, similar to the finding from this research group (19%), some participants also reported weight loss (18.2%) in our sample population. Although we did not measure the composition of dietary intake, we found that the weight gainers engaged in riskier nutritional behaviors and were less active during the peak-lockdown. Some of these risky behaviors were maintained after the lockdown eased. We also report that weight gain during the peak-lockdown was frequently followed up by continued self-reported weight gain in some participants after the lockdown eased. In other cases, self-reported weight gain during the lockdown was followed up by weight maintenance. Only rarely was weight gain during lockdown associated with weight loss after the peak lockdown eased. Overall, our results indicate that the COVID-19 lockdown periods disrupted weight management among many Americans and that associated health effects are likely to persist.

Participants who gained body weight during peak-lockdown engaged in a frequent consumption of snacks, which is a dietary behavior typically associated with excess body weight [23]. This finding is supported by a recent report from the Hunter Food study that collected self-reported data on food preferences and eating behaviors during COVID-19 from 1005 US adults [24]. Specifically, their data show that 46% of participants reported increased snacking throughout the day during the pandemic. Simultaneously, our weight gain groups also reported an increased frequency of both ultra-processed foods and fruit intake during this restriction period. While we did not measure what snacks were frequently consumed by weight gainers, we speculate that they included a large proportion of energy-dense ultra-processed foods and a low intake of healthy fruits. This snacking decreased post-lockdown, indicating a correction of action and possible motivation to improve dietary intake.

In line with other studies [25,26,27], our weight gain groups also reported high levels of boredom and stress combined with lower control on cravings and increased cravings for sweet and savory foods during the peak-lockdown. Not surprisingly, these psychological variables of boredom [28] and chronic stress [29] are known to exacerbate craving for and intake of unhealthy foods and snacks. Boredom encourages people to seek sensation [30], possibly making foods high in sugar and fat particularly appealing as potent distractors of self-regulation, while stress alters metabolism in favor of increased appetite and cravings [31]. Thus, it is plausible that the reduced inhibitory control on cravings and frequent intake of ultra-processed food was driven by boredom and stress. Our weight gain group continued to consume ultra-processed foods after the lockdown. Not surprisingly, this group also continued to report a high level of stress and lack of control over cravings. Overall, our participants exhibited less frequent eating away-from behavior (including takeout, fast food, and delivery) during the peak-lockdown period. However, when comparing weight change categories, weight gain risk groups reported higher consumption of takeout/fast food during the peak-lockdown period. Our follow-up data show that this group difference persisted after the lockdown eased. Given that away-from-home foods tend to be high in calories, fat, and sodium, but low in fruits, vegetables, and whole grains [32], this return to (or increase) in eating out behavior is of great concern. Specifically, we speculate that this behavior will interfere with the return to pre-COVID weight, particularly for high weight gain groups.

Individuals gaining body weight during the lockdown also engaged in sedentary behaviors in the form of an extended amount of time spent watching TV per day and engaging in leisure screen activity. This disproportionately high sedentary activity in weight gain groups can be attributed to restructured workdays and social activities that would typically occur outside the home. Additionally, we speculate that boredom during the lockdown may also have contributed to this behavior. With increased boredom, people gravitate toward tasks that require less cognitive load, such as the use of smartphones, the internet, or online socializing [33,34]; this may explain the relationship observed in our studies. We also report a trend in decreasing weekly energy expenditure along with an increase in body weight during the peak-lockdown. With people staying indoors (in some cities and states), the closures of many local parks and recreation areas, and cancellations of gyms and organized sports activities, these findings are not surprising and supported by other studies [35,36]. Moreover, increasing PA can reduce cravings for high-calorie foods as well as mood [37]. Thus, lower PA possibly contributed to boredom and lower control over cravings, contributing to weight gain.

The COVID-19 lockdown also provided an opportunity for a smaller percentage of people to lose weight. Individuals who lost weight reported higher stress and boredom but also reported higher levels of PA, in combination with relatively healthy nutrition behaviors (e.g., less alcohol, less snacking, less processed foods relative to other groups). These data suggests that while stress and boredom were prevalent in all weight change groups during the peak-lockdown, people who lost weight were possibly counteracted the negative impact by increasing their energy expenditure and not indulging in weight gain-promoting dietary behavior. Moreover, some participants reported relatively little disruption to weight management from the COVID-19 lockdowns. They maintained their weight and reported relatively low (compared to other groups) stress and boredom.

The results of this study should be interpreted in light of several limitations. First, while this study was longitudinal but non-experimental, causality cannot be inferred. Second, due to the nature of data collected, our results may be subject to self-reporting bias and/or recall bias. Additionally, despite the diversity of our sample and the number of participants who completed the follow-up survey, a convenience sampling approach was used, which may limit generalizability. Moreover, the degree of shelter-in-place guidelines and the number of COVID-19 cases in the participants’ area of residence likely differed, creating differences in flexibility with stepping outside the house. Finally, because COVID-19 is a pervasive and complex social context, we are unable to compare participant responses to what would have happened outside of the COVID-19 pandemic. Nor are we able to isolate aspects of the pandemic (e.g., lockdowns, personal behavior change, telecommuting, changes in work–life balance) that are driving the health behaviors changes we observed. Additionally, the role of alterations in socioeconomic and demographic factors such as a change in income, smaller personal space at home, loss of employment on psychological health, eating behaviors, and physical activity behaviors was not tested in our dataset. Such changes have been shown to increase anxiety, emotional eating, and lower PA [38,39] during the lockdown, and thus are critical to explore in future analyses. 

Altogether, this is the first study to report the longitudinal fluctuations in health and psychological factors in US adults who gained or lost weight during the peak of lockdown. Our weight change data indicate an average gain of 0.62 kg that is about half of the reported annual weight gain of 1 kg. We speculate that this pandemic weight gain combined with the average holiday weight gain of 0.5 kg will have an even greater impact on annual weight change in 2020. Overall, our results indicate that although the lockdown period was short-lived, the effects of this disruption to daily life could affect longer-term management of weight-related behaviors, putting people at greater risk of COVID-related weight gain.

## Figures and Tables

**Table 1:** Self-reported changes in behaviors during the peak-lockdown period and at 5-month follow-up during the post-lockdown period.

Behavioral Variables	Peak-Lockdown Period (March–May)	Post-Lockdown Period May–September)
Body Weight (kg) *	76.70 (19.79)	77.32 (20.01)
Body Mass Index (kg/m^2^) **	26.12 (5.81)	26.38 (5.98)
Diet variables		
Takeout/Restaurant Frequency ***	2.94 (1.41)	3.36 (1.31)
Alcohol Frequency	4.11 (1.43)	4.01 (1.28)
Vegetable Frequency	5.83 (1.43)	5.88 (1.40)
Fruit Frequency	5.62 (1.52)	5.48 (1.52)
Non-diet drinks (all SSBs) Frequency	3.50 (2.34)	3.54 (2.24)
Diet soda or other diet drinks Frequency	2.47 (2.14)	2.55 (2.15)
Processed foods (NOVA) Frequency *	4.66 (1.80)	4.52 (1.59)
Ultra-processed foods (NOVA) Frequency	3.79 (1.76)	3.71 (1.70)
Physical activity variables		
Weekly MET ***	2100.66 (2287.28)	2712.71 (2898.18)
Walking	644.93 (856.65)	647.77 (831.93)
Moderate Activity ***	482.31 (759.20)	583.98 (874.43)
Vigorous Activity	973.41 (1468.77)	953.89 (1424.85)
Sitting Time **	410.74 (290.27)	378.44 (250.90)
TV Time Frequency ***	3.87 (1.42)	3.60 (1.33)
Leisure Screen Time Frequency **	3.68 (1.40)	3.55 (1.33)
Psychological State variables		
Stress Rating	4.48 (2.59)	4.36 (2.54)
Sleep (hours)	7.21 (1.32)	7.08 (1.29)
Sleepiness Rating	2.78 (1.42)	2.76 (1.39)
Boredom Rating ***	3.49 (1.52)	3.24 (1.53)
Self-Weighing Frequency ***	2.48 (1.33)	2.62 (1.19)
Importance of Eating Healthily	2.96 (0.87)	3.00 (0.85)
Mean Cravings ***	5.78 (2.38)	6.20 (2.41)

Paired t-test was used for comparison of behaviors between two time points. Asterisks on the variable names represent *p* values, * *p* < 0.05, ** *p* < 0.01, *** *p* < 0.001. Data presented as mean (SD). NOVA = food classification system to highlight degree of processing of foods.

**Table 2:** Self-reported frequency of diet and physical activity behaviors across weight risk group during the peak lockdown (T1) and post the initial COVID-19 lockdown (T2).

		Lost Weight in March−May *N* = 134 (18.4%)	Maintained Weight in March−May *N* = 323 (44.4%)	Gained 1–4 lbs in March−May *N* = 132 (18.2%)	Gained > 5 lbsin March−May *N* = 138 (19.0%)	*p* ^#^	Time * Group Interaction
Body Mass Index (kg/m^2^)	T1	26.57 (5.62) ^ab^	25.21(5.51) ^a^	**25.58 (5.07) ^a^**	**28.32 (6.69) ^bc^**	***	9.90 ***
	T2	26.38 (5.69) ^a^	25.22(5.44) ^a^	**26.03 (5.32) ^a^**	**29.40 (7.00) ^b^**	***	
Weight (kg)	T1	77.66 (19.14) ^a^	74.41 (19.02) ^a^	**73.32 (17.78) ^a^**	**84.34 (32.00) ^b^**	***	10.18 ***
	T2	76.91 (18.92) ^a^	74.33(18.57) ^a^	**74.50 (18.44) ^a^**	**87.36 (22.53) ^b^**	***	
Diet variables Frequency							
Vegetables Frequency	T1	5.88 (1.41) ^a^	5.91 (1.46) ^a^	5.60 (1.51) ^a^	5.80 (1.27) ^a^	ns	0.83
	T2	5.88 (1.43) ^a^	6.01 (1.42) ^a^	5.67 (1.49) ^a^	5.80 (1.24) ^a^	ns	
Fruits Frequency	T1	5.68 (1.56) ^a^	5.61 (1.52) ^a^	**5.64 (1.60) ^a^**	**5.57 (1.42 ^a^**	ns	2.47
	T2	5.54 (1.49) ^a^	5.58 (1.53) ^a^	**5.30 (1.51) ^a^**	**5.33 (1.52) ^a^**	ns	
Non-diet drinks (all SSBs) Frequency	T1	3.40 (2.29) ^ab^	3.24 (2.29) ^a^	3.86 (2.35) ^ab^	3.86(2.42) ^b^	*	0.71
	T2	3.64 (2.25) ^ab^	3.28 (2.27) ^a^	3.70 (2.00) ^ab^	3.91(2.34) ^b^	*	
Diet soda or diet drinks Frequency	T1	**2.44 (2.08) ^a^**	2.37 (2.06) ^a^	2.29 (2.16) ^a^	2.91(2.31) ^a^	*	1.56
	T2	**2.75 (2.19) ^ab^**	2.37 (2.08) ^a^	2.32 (2.04) ^a^	2.99 (2.30) ^b^	*	
Processed foods (NOVA) Frequency	T1	4.40 (1.81) ^a^	4.56 (1.79) ^a^	4.95 (1.61) ^a^	4.86 (1.94) ^a^	*	ns
	T2	4.48 (1.48) ^a^	4.39 (1.81) ^a^	4.67 (1.50) ^a^	4.73 (1.73) ^a^	ns	ns
Ultra-processed foods (NOVA) Frequency	T1	3.60 (1.84) ^a^	3.63 (1.68) ^a^	3.98 (1.72) ^ab^	4.17 (1.84) ^b^	**	ns
	T2	3.66 (1.79) ^a^	3.50 (1.68) ^a^	3.70 (1.50) ^a^	4.28 (1.75) ^b^	***	ns
Snacking frequency	T1	**3.38 (1.31) ^a^**	3.30 (1.32) ^a^	**2.75 (1.29) ^b^**	2.85 (1.26) ^b^	***	*
	T2	**3.12 (1.23) ^a^**	3.30 (1.22) ^a^	**3.14 (1.25) ^a^**	3.04 (1.12) ^a^	ns	*
Alcohol Frequency	T1	3.76 (1.30) ^a^	4.19 (1.50) ^b^	**4.09 (1.53) ^b^**	4.28 (1.26) ^b^	*	*
	T2	**3.12 (1.23) ^a^**	3.30 (1.22) ^a^	**3.14 (1.25) ^a^**	3.04 (1.12) ^a^	ns	*
Takeout/Restaurant Frequency	T1	**2.95 (1.38) ^a^**	**2.81 (1.41) ^a^**	**3.18 (1.42) ^a^**	**3.02 (1.42) ^a^**	ns	*
	T2	**3.53 (1.26) ^a^**	**3.14 (1.28) ^b^**	**3.50 (1.25) ^a^**	**3.55 (1.40) ^a^**	***	ns
Physical activity variables							
Weekly Kcal MET	T1	2514.65 (2627.80) ^a^	2051.19 (2191.27) ^a^	1859.81 (2174.49) ^a^	2044.82 (2231.74) ^a^	ns	0.43
	T2	3327.72 (3360.22) ^a^	2609.33 (2813.11) ^ab^	2339.12 (2270.61) ^b^	2710.61 (3077.10) ^ab^	*	
Vigorous Activity	T1	1304.84 (1798.59) ^a^	936.07 (1429.47) ^a^	854.79 (1283.79) ^a^	852.46 (1331.88) ^a^	*	0.26
	T2	1278.21 (1798.94) ^a^	957.67 (1445.37) ^ab^	768.55 (1056.40) ^b^	804.96 (1212.78) ^b^	*	
Moderate Activity	T1	480.46 (786.98) ^a^	527.55 (802.72) ^a^	399.55 (649.82) ^a^	457.39 (723.64) ^a^	ns	0.32
	T2	646.15 (976.04) ^a^	609.55 (880.78) ^a^	521.98 (843.42) ^a^	522.19 (780.55) ^a^	ns	
Walking Time	T1	729.35 (879.01) ^a^	587.56 (745.67) ^a^	605.48 (771.32) ^a^	734.97 (1067.44) ^a^	ns	2.21
	T2	778.46 (971.36) ^a^	637.31 (798.67) ^a^	629.65 (831.24) ^a^	561.96 (752.45) ^a^	ns	
Sitting Time	T1	406.38 ^a^	**406.38 ^a^**	403.40 ^a^	426.93 ^a^	ns	0.57
	T2	359.33 ^a^	**367.88 ^a^**	396.41 ^a^	404.82 ^a^	ns	
TV time	T1	3.67 (1.48) ^a^	**3.79 (1.40) ^ab^**	**4.01 (1.36) ^ab^**	**4.14 (1.42) ^b^**	*	2.74 *
	T2	3.51 (1.40) ^a^	**3.58 (1.35) ^a^**	**3.71 (1.26) ^a^**	**3.62 (1.27) ^a^**	ns	
Leisure Screen time	T1	3.67 (1.38) ^ab^	3.51 (1.37) ^a^	3.93 (1.39) ^b^	3.83 (1.44) ^ab^	*	0.78
	T2	3.63 (1.44) ^ab^	3.42 (1.27) ^a^	3.77 (1.34) ^b^	3.55 (1.33) ^ab^	ns	

Data presented as mean (SD). Each variable presented as a 4 (weight-risk group) × 2 (time) mixed ANOVA. *p* ^#^ Denotes within time group effect. Different superscript letters indicate statistical significance when testing within time group differences. All post-hoc tests were conducted in the context of the Mixed ANOVA. Simple effects of time are presented using bold/regular text. If a weight-group differed across time, their means are in bold. Unbolded means indicate no group difference across time. Omnibus effects testing group differences within a time are indicated with asterisks: * (*p* < 0.05), ** (*p* < 0.01), or *** (*p* < 0.001). Contrasts between weight-risk group at a given time are indicated with subscripts and indicate Tukey post-hoc tests *p* < 0.05. SSBs = sugar-sweetened beverages; NOVA = food classification system to highlight degree of processing of foods. Snacking frequency (higher rating = less snacking); ns = not significant.

**Table 3:** Self-reported psychological state variables across weight risk groups during the peak lockdown (T1) and post the initial COVID-19 lockdown (T2).

		Lost Weight in March–May *N* = 134 (18.4%)	Maintained Weight in March–May *N* = 323 (44.4%)	Gained 1–4 lbs in March–May *N* = 132 (18.2%)	Gained >5 lbs in March–May *N* = 138 (19.0%)	*p* ^#^	Time * Group Interaction
Stress rating	T1	4.61 ^a^	3.87 ^b^	**4.73 ^a^**	4.92 ^a^	***	1.60
	T2	4.44 ^ab^	3.99 ^b^	**5.12** ^a^	5.07 ^a^	***	
Sleepiness rating	T1	2.72 (1.43) ^ab^	2.54 (1.39) ^b^	3.02 (1.36) ^ac^	**3.18 (1.44)** ^c^	***	3.30 *
	T2	2.57 (1.38) ^a^	2.63 (1.38) ^a^	3.11 (1.49) ^b^	**2.89 (1.23)** ^ab^	**	
Sleep time (h)	T1	7.03 (1.40) ^a^	**7.28 (1.15)** ^a^	7.14 (1.44) ^a^	**7.31 (1.48)** ^a^	ns	1.27
	T2	7.02 (1.38) ^a^	**7.10 (1.23)** ^a^	7.05 (1.49) ^a^	**7.10 (1.20)** ^a^	ns	
Boredom rating	T1	3.72 (1.48) ^a^	3.16 (1.55) ^b^	3.78 (1.36) ^a^	3.78 (1.51) ^a^	***	0.41
	T2	3.40 (1.49) ^a^	2.92 (1.55) ^b^	3.63 (1.50) ^b^	3.49 (1.43) ^b^	***	
Craving Control rating	T1	6.00 (2.12) ^a^	**6.39 (2.26) ^a^**	**5.16 (2.20) ^b^**	**4.53 (2.50) ^b^**	***	0.77
	T2	6.27 (2.33) ^a^	**6.87 (2.20) ^ab^**	**5.83 (2.37) ^ab^**	**4.95 (2.41) ^c^**	***	
Sweet Cravings rating	T1	3.99 (2.21) ^ab^	3.46 (2.21) ^b^	4.64 (2.17) ^ac^	4.76 (2.30) ^c^	***	0.80
	T2	3.66 (2.21) ^a^	2.94 (2.16) ^b^	3.83 (2.00) ^a^	4.14 (2.40) ^a^	***	
Savory Cravings rating	T1	4.16 (1.90) ^a^	**4.07 (2.08) ^a^**	**4.47 (1.89) ^ab^**	5.03 (2.10) ^b^	***	1.22
	T2	4.12 (1.89) ^a^	**3.65 (2.05) ^a^**	**3.99 (2.02) ^a^**	4.81 (2.09) ^b^	***	
Positive Mood rating	T1	5.42 (1.90) ^a^	6.10 (2.07) ^b^	5.24 (1.89) ^a^	5.29 (2.20) ^a^	***	0.55
	T2	5.72 (2.02) ^a^	6.30 (2.09) ^b^	5.41 (1.94) ^a^	5.65 (2.22) ^a^	***	
Daily self-weighing frequency	T1	2.78 (1.44) ^a^	**2.40 (1.26) ^b^**	2.45 (1.34) ^b^	2.43 (1.33) ^b^	*	0.39
	T2	2.88 (1.16) ^a^	**2.58 (1.19) ^a^**	2.52 (1.19) ^a^	2.54 (1.17) ^a^	*	

Data presented as mean (SD). Each variable presented as a 4 (weight-risk group) × 2 (time) mixed ANOVA. *p* ^#^ Denotes within time group effect. Different superscript letters indicate statistical significance when testing within time group differences. All post-hoc tests were conducted in the context of the Mixed ANOVA. Simple effects of time are presented using bold/regular text. If a weight-group differed across time, their means are in bold. Unbolded means indicate no group difference across time. Omnibus effects testing group differences within a time are indicated with asterisks: * (*p* < 0.05), ** (*p* < 0.01) or, *** (*p* < 0.001). Contrasts between weight-risk group at a given time are indicated with subscripts and indicate Tukey post-hoc tests *p* < 0.05.

## Data Availability

The data presented in this study are available upon request by the corresponding author.

## Abbreviations

List of Abbreviations:

BMI	Body mass index
Mturk	Amazon Mechanical Turk
PA	Physical Activity
SSS	Stanford Sleepiness Scale
CoEQ	The Control of Eating Questionnaire
